# Emotion Recognition Based on a EEG–fNIRS Hybrid Brain Network in the Source Space

**DOI:** 10.3390/brainsci14121166

**Published:** 2024-11-22

**Authors:** Mingxing Hou, Xueying Zhang, Guijun Chen, Lixia Huang, Ying Sun

**Affiliations:** 1 College of Integrated Circuits, Taiyuan University of Technology, Taiyuan 030600, China; houmingxing0072@link.tyut.edu.cn; 2 College of Computer Science and Technology, Taiyuan Normal University, Taiyuan 030619, China; 3 College of Electronic Information Engineering, Taiyuan University of Technology, Taiyuan 030600, China; chenguijun@tyut.edu.cn (G.C.); huanglixia@tyut.edu.cn (L.H.); sunying@tyut.edu.cn (Y.S.)

**Keywords:** emotion recognition, EEG–fNIRS, source space, brain network

## Abstract

**Background/Objectives**: Studies have shown that emotion recognition based on electroencephalogram (EEG) and functional near-infrared spectroscopy (fNIRS) multimodal physiological signals exhibits superior performance compared to that of unimodal approaches. Nonetheless, there remains a paucity of in-depth investigations analyzing the inherent relationship between EEG and fNIRS and constructing brain networks to improve the performance of emotion recognition. **Methods**: In this study, we introduce an innovative method to construct hybrid brain networks in the source space based on simultaneous EEG-fNIRS signals for emotion recognition. Specifically, we perform source localization on EEG signals to derive the EEG source signals. Subsequently, causal brain networks are established in the source space by analyzing the Granger causality between the EEG source signals, while coupled brain networks in the source space are formed by assessing the coupling strength between the EEG source signals and the fNIRS signals. The resultant causal brain networks and coupled brain networks are integrated to create hybrid brain networks in the source space, which serve as features for emotion recognition. **Results**: The effectiveness of our proposed method is validated on multiple emotion datasets. The experimental results indicate that the recognition performance of our approach significantly surpasses that of the baseline method. **Conclusions**: This work offers a novel perspective on the fusion of EEG and fNIRS signals in an emotion-evoked experimental paradigm and provides a feasible solution for enhancing emotion recognition performance.

## 1. Introduction

Emotion is an expression of human intelligence, exerting a crucial influence on social interactions. Emotion recognition is a key issue in affective computing and is attracting increasing attention in the field of artificial intelligence [1,2,3], which is widely used in many domains, such as human–computer interaction, intelligent education, transportation safety, and healthcare. Physiological signals, regulated by the nervous system, are inherently difficult to conceal and disguise, rendering recognition based on these signals more dependable than that derived from non-physiological signals such as facial expressions, body postures, and voice tones [4]. Electroencephalogram (EEG), which captures the electrical field caused by neural activity through electrodes placed on the scalp [5], has been extensively explored in emotion recognition by virtue of its high temporal resolution, as well as its non-invasive and low-cost characteristics. Meanwhile, functional near-infrared spectroscopy (fNIRS) measures the concentration change of oxygenated hemoglobin (HbO) and deoxygenated hemoglobin (HbR) associated with brain activities through optodes (transmitter and receiver) positioned on the scalp, which reflects the hemodynamic activity of the cerebral cortex and boasts high spatial resolution and robust interference resistance. Consequently, fNIRS effectively mitigates the spatial resolution limitations of EEG signals and furnishes supplementary insights into neuronal activity [6]. As a result, research on emotion recognition based on the joint analysis of simultaneous EEG–fNIRS signals is garnering interest among researchers.

In current studies on EEG-based emotion recognition, researchers have proposed many features centered on the intrinsic attributes of EEG signals, including temporal, spectral, time–frequency, and spatial features [7,8,9,10]. However, these features cannot characterize the information transmission and interaction between brain regions during emotional cognition. To address this issue, researchers proposed EEG brain network features, which facilitated the examination of coordination mechanisms among brain regions from a macroscopic perspective and enhanced the performance of emotion recognition [11,12,13,14,15]. An EEG brain network is constructed by treating EEG signals as nodes and the connectivity between nodes as edges. Depending on the connectivity metrics, brain networks are categorized into functional brain networks, established by undirected metrics, such as correlation and mutual information, and effective brain networks, constructed through directed measures like transfer entropy and Granger causality [16]. Effective brain networks not only reflect the interactions between nodes, but also indicate the information flow direction, thereby elucidating the information exchange pattern among brain regions. Granger causality (GC) analysis, with no prior information required, could calculate the causal relationship between time series, which was then used to construct the EEG causal brain networks, garnering some achievements in emotion recognition [17,18,19,20]. Nevertheless, existing EEG-based causal brain networks have overlooked the volume conduction effect inherent in EEG signals [21], which reduced the precision in reflecting the information transmission pattern across brain regions under various emotions, thus constraining further improvements in emotion recognition performance. To mitigate the impact of the volume conduction effect, researchers have applied the source localization technique to EEG signals, yielding EEG source signals that represent cortical electrical activity more accurately, and have conducted subsequent analyses in the source space. Chen et al. [22] extracted six temporal and spectral features from EEG source signals for emotion recognition, realizing notable improvements compared to the features derived from EEG signals. Similarly, Becker et al. [23] extracted high-order cross features, statistical features, and spectral features from both EEG source signals and EEG signals for emotion recognition, further substantiating the superiority of features from EEG source signals. In this paper, we propose to construct more precise causal brain networks from EEG source signals using Granger causality analysis, termed as causal brain networks in the source space, which hold significant promise for advancing the performance of emotion recognition.

Additionally, owing to the good compatibility and complementarity, EEG and fNIRS multimodal fusion has become a research hotspot in many fields [24,25,26,27]. In recent years, the joint analysis of EEG–fNIRS for emotion recognition has also drawn much attention from researchers. Currently, the absence of publicly available emotional datasets containing concurrent EEG–fNIRS signals has limited research to a select few who have conducted preliminary investigations using self-built datasets. These studies have demonstrated that emotion recognition utilizing EEG–fNIRS multimodal signals outperforms recognition performed using unimodality [28,29,30,31]. Nevertheless, the existing EEG–fNIRS fusion methods are mainly confined to data-level or feature-level fusion by machine learning models, wherein features are independently extracted from each modality. There remains a lack of deep exploration into the intrinsic relationship between EEG and fNIRS signals. Therefore, the pursuit of a novel EEG–fNIRS fusion method for emotion recognition is of profound significance.

Research on the intrinsic relationship between EEG and fNIRS is anchored in the concept of neurovascular coupling, a well-regulated physiological process wherein neural activity in the brain is inherently accompanied by fluctuations in blood flow [32]. In particular, upon neuronal activation, blood flow is directed towards the active region to satisfy the heightened demand for glucose and oxygen, thereby inducing detectable fluctuations in hemoglobin concentration through fNIRS. This phenomenon reflects the close relationship between neuronal activity and hemodynamic changes in the brain, providing a new perspective for EEG–fNIRS fusion. Current studies on the coupling relationship between EEG and fNIRS primarily concentrate on motor imagery tasks, where the experimental stimuli of short duration and constant intensity can be modeled as square wave functions carried by EEG information. By convolving the modeling function with the canonical hemodynamic response function (HRF), the predicted fNIRS signal is obtained and used as a design matrix for fitting the measured fNIRS signals within a general linear model (GLM), yielding regression coefficients that reflect the EEG–fNIRS coupling relationship. Li et al. [33] employed event-related potentials (ERPs) from specific frequency EEG signals to model the experimental stimuli, while Gao et al. [34] modeled the experimental stimuli using the peak and latency of the time-varying power of the channel-averaged EEG signal. However, in the emotion-evoked experimental paradigm, the stimuli are generally in the form of audio or video clips, with relatively prolonged duration (1–2 min) and continuously changing intensity, precluding their exact description by square wave functions. Therefore, this paper introduces a new method for modeling the stimuli in an emotion-evoked experimental paradigm. By leveraging the neurovascular coupling characteristic, the coupling strength between the EEG source signal and the fNIRS signal is calculated, serving as a new connectivity metric to construct a coupled brain network in the source space for emotion recognition.

In summary, in this paper, we propose an innovative method for constructing hybrid brain networks in the source space from concurrent EEG–fNIRS signals for emotion recognition. First, we impose source localization on EEG signals to gain more precise cortical electrical activity, termed EEG source signals, aiming at alleviating the impact of the volume conduction effect. Then, Granger causality analysis is performed on the EEG source signals to construct causal brain networks in the source space. Furthermore, according to the neurovascular coupling characteristic, we introduce a novel approach for calculating coupling strengths between EEG source signals and fNIRS signals, thereby constructing coupled brain networks in the source space under an emotion-evoked experimental paradigm. Finally, by merging the two brain networks, hybrid brain networks are generated in the source space, making the most of the causal relationship among the EEG source signals and the coupling relationship between the EEG source signals and fNIRS signals to promote emotion recognition performance.

The main contributions are summarized as follows:

(1) A novel EEG–fNIRS fusion method for constructing coupled brain networks in an emotion-evoked experimental paradigm is proposed.
(2) Emotion recognition based on hybrid brain networks, achieved by integrating causal brain networks and coupled brain networks in the source space, is explored for the first time in this paper.
(3) Evaluations on our self-built dataset (ENTER) and public datasets (SEED-IV, DEAP) show the superior performance of the proposed method.

The rest of the paper is organized as follows. Section 2 introduces EEG–fNIRS data acquisition and preprocessing; Section 3 elaborates on the proposed method; the experimental results and analysis are presented in Section 4. Finally, Section 5 concludes the paper and suggests promising directions for future research.

## 2. Data Acquisition and Preprocessing

In this section, we provide descriptions regarding the process of data acquisition for the ENTER dataset and data preprocessing.

### 2.1. Data Acquisition

To leverage the complementary advantages of EEG and fNIRS for emotion recognition research, our team simultaneously collected EEG–fNIRS signals in an emotion-evoked experiment and built an emotional dataset, named ENTER [31]. A more detailed description of and access to the dataset is available online at https://gitee.com/tycgj/enter (accessed on 10 August 2024). The data for each subject are stored in a separate folder, with the name as the subject ID. In each folder, there are two subfolders, named “EEG” and “FNIRS”, containing EEG and fNIRS data saved as MAT files from 60 trails, respectively.

**Figure 1:**
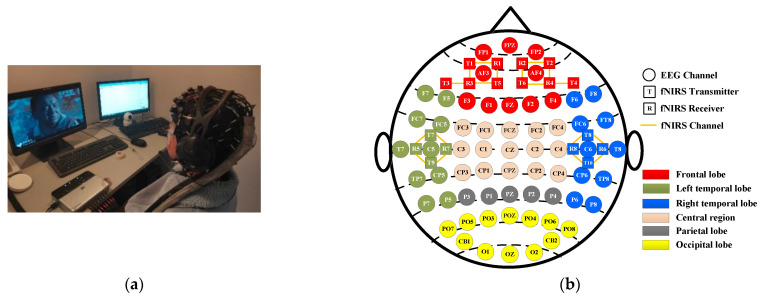
Experimental setup for EEG–fNIRS data acquisition. (**a**) Experimental scenario; (**b**) positions of EEG electrodes and fNIRS optodes.

**Emotion-inducing materials:** 60 videos (1–2 min long) were carefully selected to induce four types of emotions, including sadness, happiness, calm, and fear (there are 15 videos pertaining to each emotion).
**Subjects:** 50 college students, 25 male and 25 female, were recruited for emotion data collection. Prior to the experiment, all subjects were informed of the experimental purpose, procedures, and important notes, and all subjects provided written informed consent.
**Signal acquisition equipment:** EEG signals were acquired at 1000 Hz using the ESI NeuroScan system (Compumedics Ltd., Victoria, Australia), which comprises 62 channels placed across the entire brain region. Concurrently, a portable near-infrared brain functional imaging system, NirSmart, was used to collect fNIRS signals at 11 Hz, with 18 channels created by adjacent transmitter–receiver pairs, which are distributed only in the frontal and temporal lobes. The experimental scenario is shown in Figure 1a, and a schematic illustration of the positions of the EEG electrodes and fNIRS optodes is shown in Figure 1b.

**Figure 2:**
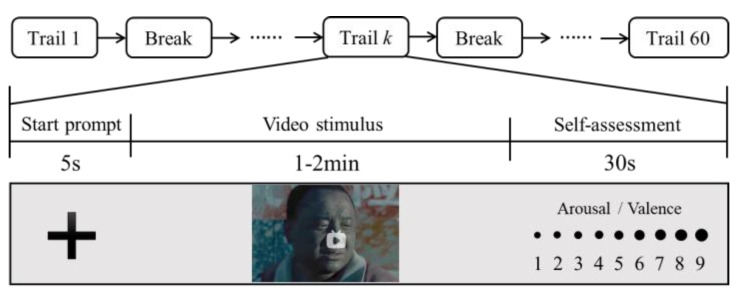
Emotion-evoked experimental paradigm.

All data acquisition experiments were executed in a screened chamber. During the experiment, a subject was seated in a comfortable chair and engaged in emotion-evoked tasks, remaining quiet and relaxed while endeavoring to minimize body movements and blinking. The experimental paradigm is shown in Figure 2. Each subject completed 60 trials. In each trial, a 5 s start prompt was followed by a continuous video clip designed to induce a specific emotion. Once the video ended, the subject was given 30 s for self-assessment, rating the emotional experience using a nine-point scale regarding arousal and valence to confirm whether the corresponding emotion was successfully induced. After a trial, the subject took a break for 2–3 min. Upon completion of all 60 trials, each subject yielded 60 sets of concurrent EEG data from 62 channels and fNIRS data from 18 channels. 

### 2.2. Data Preprocessing

The collected EEG data typically includes irrelevant signals such as ocular movement artifacts, muscle artifacts, power line noise, and electromagnetic disturbances. Therefore, data preprocessing is indispensable. The EEG signals undergo re-referencing and baseline correction, succeeded by artifact removal through independent component analysis (ICA). Thereafter, the signals are processed with a bandpass filter from 0.5 to 45 Hz and downsampled to 200 Hz. The recorded fNIRS optical density signals are subjected to baseline correction and filtered with a bandpass of 0.01–0.2 Hz. Subsequently, these signals are converted to concentration changes of HbO, according to the Modified Beer–Lambert law [35], and finally upsampled to 200 Hz to match the EEG signals.

## 3. Proposed Method

**Figure 3:**
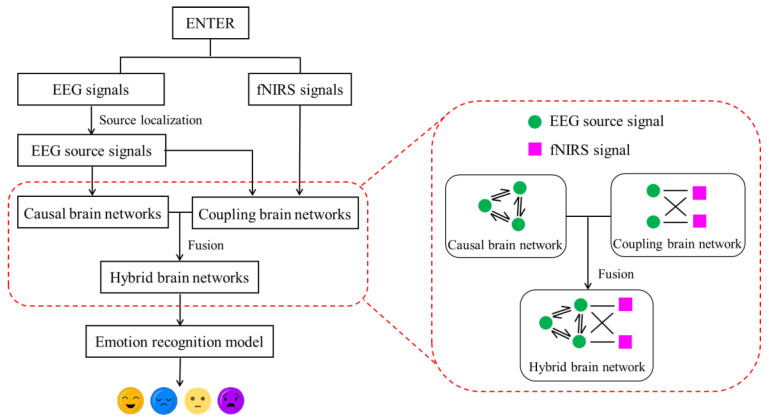
The overall flowchart of our approach.

In this paper, we propose an innovative method for constructing hybrid brain networks in the source space from concurrent EEG–fNIRS signals for emotion recognition. The overall flowchart of our approach is shown in Figure 3. First, we apply Granger causality analysis to EEG source signals, obtained through the source localization technique, to establish causal brain networks. Then, the coupling strengths between the EEG source signals and the fNIRS signals are calculated to generate coupled brain networks. Finally, we integrate causal and coupled brain networks to create hybrid brain networks in the source space, which are fed into a recognition model as features for emotion classification.

### 3.1. Causal Brain Networks Construction in the Source Space

This section depicts the source localization technique for estimating EEG source signals from EEG signals [36], as well as the process of constructing causal brain networks from EEG source signals, namely causal brain networks in the source space.

#### 3.1.1. Source Localization

When cortical electrical activities, modeled as dipoles, occur, EEG signals can be detected on the scalp. The relationship between dipoles J and EEG signals X can be described as follows:

(1) X=LJ+δ

where $X\in {\mathbb{R}}^{u\times n}$ represents EEG signals with u channels and n time samples, $J\in {\mathbb{R}}^{v\times n}$ indicates that v dipoles with n time samples exist in the cerebral cortex, δ represents noise, and $L\in {\mathbb{R}}^{u\times v}$ represents the lead field matrix, which describes the electric field generated by the unit dipole and can be calculated based on the parameters of the head model. In application, the EEG signal X is measurable; hence, J can be estimated from X, which is referred to as inverse problem. Generally, u≪v, rendering Equation (1) highly underdetermined, implies that numerous different combinations of dipoles can produce the identical electric field distribution on the scalp. Therefore, additional constraints are requisite for the solution. A prevalent approach is to minimize the residual function, as follows:

(2) $$R(J)=\alpha {\left\|X-LJ\right\|}^{2}+J^{T}\Sigma_{0}J$$

where the first term on the right side represents the reconstruction error, quantifying the difference between the measured EEG signals and those reconstructed by dipoles; the second term is regularization term used to address the underdetermined problem; $\Sigma_{0}$ is a regularization matrix; α controls the weight between the reconstruction error and the regularization term.

Different regularization matrices will produce different solutions for source localization. When $\Sigma_{0}$ is the identity matrix, the MNE (minimum norm estimate) [37] solution of Equation (1) is given by the following:

(3) $$J=\Sigma_{0}^{-1}L^{T}{\left(L\Sigma_{0}^{-1}L^{T}+\alpha^{-1}I\right)}^{-1}X$$

wherein I denotes the identity matrix.

**Figure 4:**
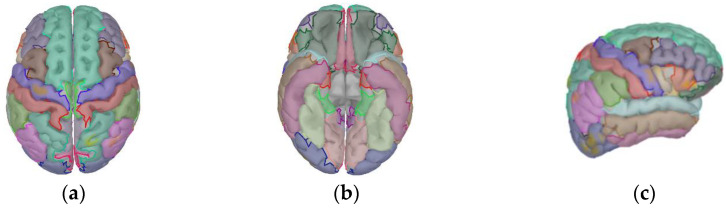
The schematic diagram of the DKT atlas. (**a**) Superior view; (**b**) basal view; (**c**) lateral view.

This study employs the MNE algorithm to determine the strengths of the dipoles and generate time series. Subsequently, the standard Desikan–Killiany–Tourville (DKT) atlas [38] is adopted to partition the cortical surface into 62 regions. The schematic diagram of the DKT atlas is shown in Figure 4.

After averaging the time series of all the dipoles within a region, an EEG source signal is generated. These EEG source signals from all regions can be represented as $S=[s_{1}(t),s_{2}(t),\cdots ,s_{c}(t)]\in {\mathbb{R}}^{n\times c}$, where c and n represent the number and sample times of the EEG source signals, respectively. It can be seen that the time resolution of the EEG and EEG source signals is consistent because of the same sample times n. However, the EEG source signals have a higher spatial resolution than does the EEG, primarily due to the reduced impact of the volume conduction effect.

#### 3.1.2. Causal Brain Networks Construction

Before constructing causal brain networks, EEG source signals are first segmented to generate samples via a 3 s rectangular window, with a contiguous sliding overlap of 1.5 s. It has been proven that the above segmentation mode is able to achieve superior performance in emotion recognition [20]. In this subsection, a sample including EEG source signals is expressed as $S^{\prime }=[{s^{\prime }}_{1}(t),{s^{\prime }}_{2}(t),\cdots ,{s^{\prime }}_{c}(t)]\in {\mathbb{R}}^{r\times c}$, where r denotes the signal length. Next, Granger causality analysis is performed on all samples to construct causal brain networks in the source space. It is worth noting that Granger causality analysis operates under the assumption of time series stationarity, meaning that the statistical attributes of a time series remain invariable over time (i.e., the mean and variance are constant). Hence, a detrending operation is necessary before conducting the analysis.

Granger causality analysis employs regression models to determine the predictive capability of one time series on another time series [39]. Specifically, the presence of a causal relationship between two time series is indicated when the inclusion of one series is beneficial for predicting the other series. To further illustrate, we suppose that two time series $s_{1}^{\prime }(t)$ and $s_{2}^{\prime }(t)$ can be described as follows:

(4) $$s_{1}^{\prime }(t)=\sum_{k=1}^{p}a_{1,k}s_{1}^{\prime }(t-k)+\xi_{1}(t)$$

(5) $$s_{1}^{\prime }(t)=\sum_{k=1}^{p}b_{1,k}s_{1}^{\prime }(t-k)+\sum_{k=1}^{p}b_{2,k}s_{2}^{\prime }(t-k)+\eta_{1}(t)$$

where t denotes time, k denotes time lag, a and b denote regression coefficients, $\xi_{1}$ and $\eta_{1}$ denote residuals (prediction errors), and p is called the model order, which denotes the maximum time lag and can be determined by Bayesian information criterion (BIC) [40].

In an autoregressive model described by Equation (4), the current observation of $s_{1}^{\prime }(t)$ is predicted by its own past observations. Conversely, in the vector regression model described by Equation (5), the current observation of $s_{1}^{\prime }(t)$ is predicted by the past observations of its own and $s_{2}^{\prime }(t)$. If the variance of $\eta_{1}$ is less than $\xi_{1}$, meaning the joining of $s_{2}^{\prime }(t)$ enhancing the prediction accuracy of $s_{1}^{\prime }(t)$, $s_{2}^{\prime }(t)$ is said to Granger-cause $s_{1}^{\prime }(t)$, expressed as $s_{2}^{\prime }\to s_{1}^{\prime }$. The magnitude of causality is quantifiable through the ratio of the variances of the residual from two models, as follows:

(6) $$F_{s_{2}^{\prime }\to s_{1}^{\prime }}=\ln\frac{\mathrm{var}(\xi_{1})}{\mathrm{var}(\eta_{1})}$$

where var(⋅) denotes variance.

After conducting a Granger causality analysis on any two EEG source signals within a sample, a causal matrix $G_{s}$ is obtained.

(7) $$G_{s}=\left[\begin{array}{cccc} g_{11} & g_{12} & \cdots & g_{1c}\\ g_{21} & g_{22} & \cdots & g_{2c}\\ \vdots & \vdots & \ddots & \vdots \\ g_{c1} & g_{c2} & \cdots & g_{cc} \end{array}\right]$$

where $g_{ij}$ represents the causality measurement of $s_{i}^{\prime }\to s_{j}^{\prime }$, indicating the direction of information transmission. Generally, $g_{ij}$ is not equal to $g_{ji}$, thereby resulting in asymmetric $G_{s}$. The pseudocode for calculating a causal matrix in the source space is provided in Algorithm 1.
**Algorithm 1:** Calculation of a causal matrix in the source space.**Input**: A sample including EEG source signals $S^{\prime }=[s_{1}^{\prime }(t),s_{2}^{\prime }(t),\cdots ,s_{c}^{\prime }(t)]\in {\mathbb{R}}^{r\times c}$**Output**: $G_{s}$ 1: **for** i=1,⋯,c, **do** 2:       **for** j=1,⋯,c, **do** 3:             Calculate the residual $\xi_{1}$ in autoregressive model by Equation (4). 4:             Calculate the residual $\eta_{1}$ in vector regression model by Equation (5). 5:             Calculate the Granger causality between the $i^{\mathrm{th}}$ and $j^{\mathrm{th}}$ EEG source sign by Equation (6). 6:       **end for** 7: **end for**

When $G_{s}$ is used as an adjacency matrix, a causal brain network in the source space can be constructed, with the $i^{\mathrm{th}}$ and $j^{\mathrm{th}}$ EEG source signals serving as nodes, and $g_{ij}$ serving as a directed edge connecting the two nodes.

### 3.2. Coupled Brain Networks Construction in the Source Space

Here, we introduce an innovative EEG–fNIRS fusion method founded on the intrinsic neurovascular coupling relationship between two signals. Given the fact that emotional intensity is fluctuating temporally, and the brain response to the same stimulus exhibits regional disparities in the emotion-evoked paradigm, we use the time-varying powers from all EEG source signals to model the experimental stimuli. Thereafter, the coupling strengths between the EEG source signals and the fNIRS signals are calculated to derive a coupling matrix, facilitating the construction of a coupled brain network in the source space.

**Figure 5:**
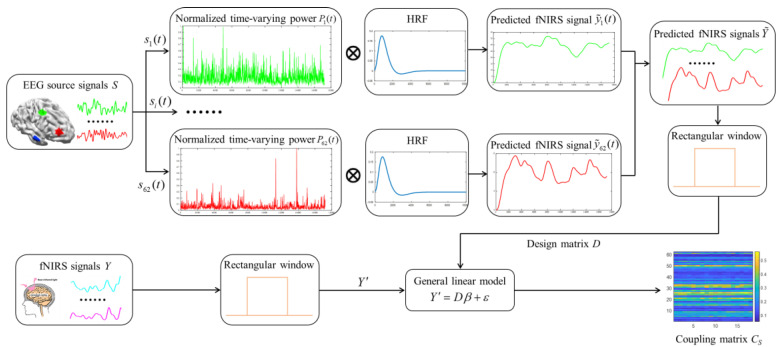
The overall process of calculating a coupling matrix.

For the EEG source signals $S=[s_{1}(t),s_{2}(t),\cdots ,s_{c}(t)]\in {\mathbb{R}}^{n\times c}$ and the measured fNIRS signals $Y=[y_{1}(t),y_{2}(t),\cdots ,y_{d}(t)]\in {\mathbb{R}}^{n\times d}$, where d denotes the number of fNIRS channels, the overall process of calculating a coupling matrix in the source space is shown in Figure 5.

Initially, we compute the time-frequency power spectrum of each EEG source signal $s_{i}(t)\in {\mathbb{R}}^{n\times 1}\;(i=1,2,\cdots ,c)$ using a short-term Fourier transform (STFT).

(8) $$P_{i}(t,f)={\left|\int_{-\infty }^{\infty }s_{i}(\tau )h(\tau -t)e^{-j2\pi ft}d\tau \right|}^{2}$$

where t denotes time; f denotes frequency; h(τ−t) denotes window function.

Following the power addition in the frequency dimension on $P_{i}(t,f)$ and normalization, the normalized time-varying power $P_{i}(t)$ is obtained and deemed as the $i^{\mathrm{th}}$ modeling signal for the corresponding experimental stimulus.

(9) $$P_{i}(t)=\mathrm{norm}(\sum_{f=f_{\min}}^{f_{\max}}P_{i}(t,f))$$

where $f_{\max}$ and $f_{\min}$ denote the upper and lower limit of the frequency range of the EEG source signal; norm(⋅) denotes normalization operator.

Subsequently, $P_{i}(t)$ is convolved with the canonical hemodynamic response function (HRF) to derive the predicted fNIRS signal ${\tilde{y}}_{i}(t)$ for the $i^{\mathrm{th}}$ modeling signal, which can be described as follows:

(10) $${\tilde{y}}_{i}(t)=P_{i}(t)⊗HRF(t)$$

Take the first n values to derive ${\tilde{y}}_{i}(t)\in {\mathbb{R}}^{n\times 1}$ to ensure the aligned signal length. The expression of HRF(t) is as follows:

(11) $$HRF(t)=g(t,6)-\frac{1}{6}g(t,16)$$

where

(12) $$g(t,d)=\frac{t^{d-1}e^{-t}}{\Gamma (d)}$$

When all ${\tilde{y}}_{i}(t)$ are obtained, we create a matrix $\tilde{Y}=[{\tilde{y}}_{1}(t),\;{\tilde{y}}_{2}(t),\cdots ,{\tilde{y}}_{c}(t)]\in {\mathbb{R}}^{n\times c}$ to represent all the predicted fNIRS signals of the current experimental stimulus. Whereafter, we segment both the predicted and measured fNIRS signals into l samples using a slide rectangular window of 3 s with an overlap of 1.5 s. Next, the coupling strengths are computed on all samples to construct coupled brain networks in the source space. Specifically, for a sample including the predicted fNIRS signals ${\tilde{Y}}^{\prime }=[{\tilde{y}}_{1}^{\prime }(t),\;{\tilde{y}}_{2}^{\prime }(t),\cdots ,{\tilde{y}}_{c}^{\prime }(t)]\in {\mathbb{R}}^{r\times c}$ and the measured fNIRS signals $Y^{\prime }=[y_{1}^{\prime }(t),y_{2}^{\prime }(t),\cdots ,y_{d}^{\prime }(t)]\in {\mathbb{R}}^{r\times d}$, ${\tilde{Y}}^{\prime }$ is used as the design matrix D to fit $Y^{\prime }$ within a general linear model, which can be formulated as follows:

(13) $$Y^{\prime }=D\beta +\varepsilon =\left[\begin{array}{ccc} {\tilde{y}}_{11}^{\prime } & \cdots & {\tilde{y}}_{1c}^{\prime }\\ \vdots & \cdots & \vdots \\ {\tilde{y}}_{r1}^{\prime } & \cdots & {\tilde{y}}_{rc}^{\prime } \end{array}\right]\left[\begin{array}{ccc} \beta_{11} & \cdots & \beta_{1d}\\ \vdots & \cdots & \vdots \\ \beta_{c1} & \cdots & \beta_{cd} \end{array}\right]+\left[\begin{array}{ccc} \varepsilon_{11} & \cdots & \varepsilon_{1d}\\ \vdots & \cdots & \vdots \\ \varepsilon_{r1} & \cdots & \varepsilon_{rd} \end{array}\right]$$

where $\beta \in {\mathbb{R}}^{c\times d}$ represents the fitting coefficient matrix; ε represents the fitting error. Since the $i^{\mathrm{th}}$ column of design matrix D contains information from the $i^{\mathrm{th}}$ EEG source signal, the element $\beta_{ij}$ of β represents the coupling coefficient between the $i^{\mathrm{th}}$ EEG source signal and the $j^{\mathrm{th}}$ fNIRS signal. 

Ultimately, we take the absolute value of the coupling coefficient as the coupling strength. Thus, the EEG–fNIRS coupling matrix $C_{S}$ in the source space can be described as follows:

(14) $$C_{S}=\left|\beta \right|$$

where $\left|\beta_{ij}\right|$ represents the coupling strength between the $i^{\mathrm{th}}$ EEG source signal and the $j^{\mathrm{th}}$ fNIRS signal. 

The pseudocode for calculating a coupling matrix in the source space is given in Algorithm 2.
**Algorithm 2:** Calculation of a coupling matrix in the source space.**Input**: EEG source signals $S=[s_{1}(t),s_{2}(t),\cdots ,s_{c}(t)]\in {\mathbb{R}}^{n\times c}$ and measured fNIRS signals $Y=[y_{1}(t),y_{2}(t),\cdots ,y_{d}(t)]\in {\mathbb{R}}^{n\times d}$**Output**: $C_{S}$ 1: **for** i=1,⋯,c, **do** 2:       Calculate the time-frequency power spectrum $P_{i}(t,f)$ for $s_{i}(t)$ by Equation (8). 3:       Calculate the normalized time-varying power $P_{i}(t)$ for $s_{i}(t)$ by Equation (9). 4:       Calculate the predicted fNIRS signal ${\tilde{y}}_{i}(t)$ by Equation (10). 5: **end for** 6: Create matrix $\tilde{Y}$ by utilizing all ${\tilde{y}}_{i}(t)$. 7: Segment $\tilde{Y}$ and Y into l samples; each sample contains ${\tilde{Y}}^{\prime }$ and $Y^{\prime }$. 8: **for** i=1,⋯,l, **do** 9:          Fit $Y^{\prime }$ within general linear model by Equation (13). 10:       Calculate the coupling matrix in the source space $C_{S}$ by Equation (14). 11: **end for**

When $C_{S}$ is used as an adjacency matrix, an EEG–fNIRS coupled brain network in the source space can be constructed, with the $i^{\mathrm{th}}$ EEG source signal and the $j^{\mathrm{th}}$ fNIRS signal serving as nodes and $\left|\beta_{ij}\right|$ serving as the edge connecting the two nodes.

### 3.3. Hybrid Brain Networks Construction in the Source Space

By concatenating the EEG causal brain network $G_{S}$ and the EEG–fNIRS coupled brain network $C_{S}$, it is feasible to derive a hybrid brain network in the source space, as follows:

(15) $$H_{S}=\mathrm{concat}(G_{S},C_{S})$$

where concat(⋅) represents the matrix concatenation. The hybrid brain network in the source space perfectly integrates the causal brain network and the coupled brain network by treating EEG source signals as intermediate nodes, not only considering the information interaction among the brain regions during emotional cognition but also encompassing the intrinsic relationship between two signals associated with neuronal activity in the brain, providing rich emotional information to enhance recognition performance.

During emotion recognition, each matrix representing a hybrid brain network is vectorized to constitute the feature vector. Combining the feature vectors of all samples, we can obtain a feature set, which will be employed to train and evaluate the emotion recognition model.

## 4. Experimental Results and Analysis

This section will present abundant experimental results to validate the effectiveness of the proposed method from two aspects: performance evaluation and performance comparison. All experiments were conducted on MATLAB R2023b. To mitigate the influence of dataset partitioning, a five-fold cross-validation strategy was adopted. The mean classification accuracy per subject from five repetitions was designated as the subject-specific recognition result. The average recognition result across 50 subjects was determined as the final emotion recognition accuracy. Furthermore, the experimental outcomes were compared with other methods extant in the literature to demonstrate the superiority of the proposed method.

### 4.1. Performance Evaluation

We first conduct a comparative analysis of the emotion recognition accuracy by utilizing different brain network features on our self-built emotion dataset ENTER, including the following: (1) EG: EEG Granger causal brain network; (2) SG: Granger causal brain network in the source space, described by $G_{S}$; (3) EC: EEG–fNIRS coupled brain network; (4) SC: coupled brain network in the source space, described by $C_{S}$; (5) EG_EC: hybrid brain network created by integrating EG and EC; (6) SG_SC: hybrid brain network $H_{S}$ created by integrating SG and SC. To eliminate the impact of a particular classifier on recognition performance, two classifiers, i.e., support vector machine (SVM), with a linear kernel, and k-nearest neighbor (KNN), with a predefined 10 neighbors, are utilized for emotion classification. Emotion recognition accuracies (%) based on different brain network features are listed in Table 1.

From Table 1, it can be observed that for SVM, the accuracy of SG achieves an 8.1% improvement over that of EG; meanwhile, the accuracy of SC outperforms the EC network by 4.8%. A similar situation regarding improvements can also be observed when KNN is used. Moreover, when we use the hybrid brain network for emotion recognition, both SVM and KNN exhibit a consistent trend where the accuracies of EG_EC surpass the results of EG or EC, and the accuracies of SG_SC show a marked improvement over the performance of SG or SC. Moreover, we also observe that the brain networks constructed in the source space (SG, SC, SG_SC) all achieve lower recognition standard deviations than their corresponding counterparts (EG, EC, EG_EC). These findings suggest that (1) constructing brain networks in the source space greatly contributes to the promotion of recognition performance; (2) the EEG–fNIRS hybrid brain networks can achieve superior recognition performance over that of causal or coupled brain networks. Notably, the results comparison shows that the proposed method (SG_SC) achieves the highest recognition accuracy and the lowest standard deviation (96.6 ± 2.08% for SVM and 91.7 ± 3.02% for KNN), verifying the superiority of our method.

**Figure 6:**
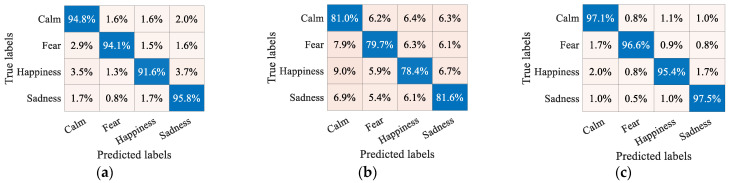
SVM recognition confusion matrices of three brain networks. (**a**) SG; (**b**) SC; (**c**) SG_SC.

To provide insight into the recognition results across different emotions, we present the SVM recognition confusion matrices for three brain networks (SG, SC, and SG_SC) in Figure 6. As shown in Figure 6a, the SG-based results reveal that the results for happiness exhibit the lowest accuracy (91.7%), with the highest misclassification rate of 3.7% for the misidentification of happiness as sadness, manifesting the relatively poor ability of SG to distinguish between sadness and happiness. The SC-based results presented in Figure 6b show a similar trend, with the results for happiness again presenting the lowest recognition accuracy of 78.4%. The highest misclassification rate of 9.0% towards calm suggests the limited discriminative power of SC between happiness and calm. The above findings indicate a heightened susceptibility of happiness to misclassification and the discrepancy between the ability of SG and SC to distinguish between different emotions. By integrating SG and SC, the proposed SG_SC greatly diminishes the inter-emotional confusion, where the highest misclassification rate of happiness is reduced to 2.0%, as shown in Figure 6c, demonstrating an excellent discriminative capability by capitalizing on the respective advantages of each.

**Figure 7:**
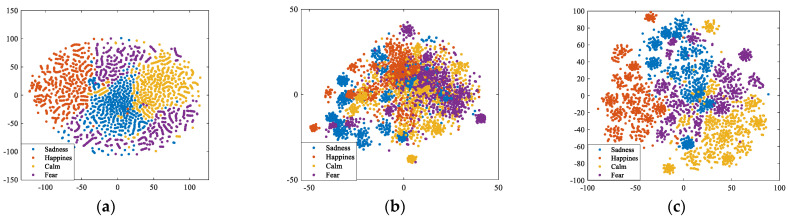
Samples distribution in 2-D feature space. (**a**) SG; (**b**) SC; (**c**) SG_SC.

To intuitively explore the emotion discrimination ability of different brain network features, we further visualize the sample distribution of SG, SC, and SG_SC in 2-D feature space using *t*-distributed stochastic neighbor embedding (*t*-SNE), as shown in Figure 7. From Figure 7a, we observe that for SG, the discrimination among emotions is evident, albeit with relatively sparse intra-class distances. Conversely, as depicted in Figure 7b, SC shows a compact intra-class distribution, although it is accompanied by severe overlap among emotions, resulting in lower inter-class distinction. These findings manifest the obvious complementarity of SG and SC. In Figure 7c, SG_SC, despite retaining some inter-class overlap, obviously achieves a more compact intra-class distribution compared to that of SG and a superior inter-class distinction relative to SC, which further confirms the effectiveness of our method in promoting emotion recognition performance.

### 4.2. Performance Comparison

In this subsection, we validate the advantages of the proposed method from two perspectives: recognition performance in different datasets and comparison with existing methods.

#### 4.2.1. Recognition Performance in Different Datasets

Since there are no publicly available emotion dataset that include concurrent EEG and fNIRS signals, except our self-built ENTER, we only take the proposed SG constructed from EEG source signals for performance validation on three emotion datasets: ENTER, SEED-IV [41], and DEAP [42]. The SEED-IV dataset is an EEG emotional dataset comprised of 15 subjects, with emotions including happiness, sadness, fear, and neutral. For each subject, three sessions were conducted on different days, and each session contained 24 trials. In one trial, EEG signals were recorded through 62 channels while the subject watched a film clip. The DEAP dataset consists of EEG signals and peripheral physiological signals from 32 subjects in regards to four emotions: high arousal-high valence (HAHV), high arousal-low valence (HALV), low arousal-high valence (LAHV), and low arousal-low valence (LALV). Every subject completed 60 trials, and the EEG signals were recorded through 32 channels. Owing to the fact that no fNIRS data are contained in the SEED-IV and DEAP datasets, we only used the EEG signal to validate the recognition performance of SG in this paper. Table 2 lists the detailed information for the three datasets.

**Figure 8:**
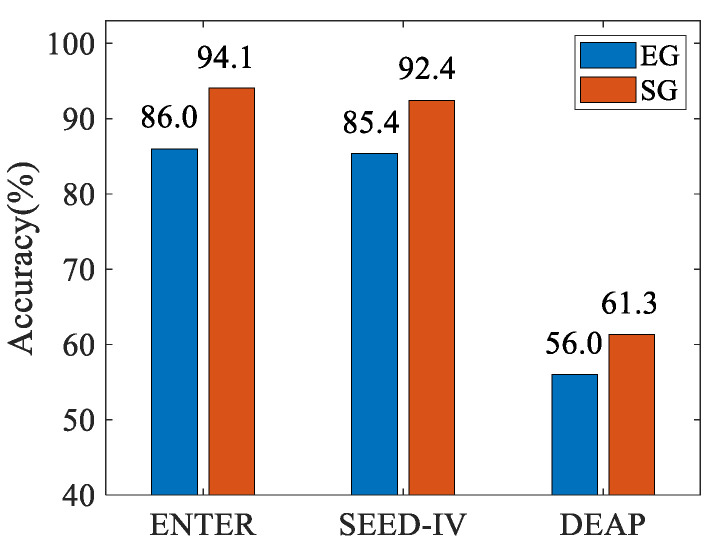
Emotion recognition accuracies (%) of SG and EG from three datasets.

The emotion recognition accuracies of SG and its counterpart EG for the three datasets are illustrated in Figure 8. From the results, we can clearly observe a consistent superiority of SG-based emotion recognition over those based on EG for all three datasets. These comparative results successfully affirm the effectiveness of the proposed SG feature extracted from EEG source signals rather than for EG extracted from EEG signals. The poor performance of both EG and SG in the DEAP dataset may result from its fewer EEG channels, which only provide limited emotional information that is not adequate to support accurate source localization.

#### 4.2.2. Comparison with Existing Methods

To further highlight the performance superiority, we conducted comparative experiments comparing our proposed features with the following: differential asymmetry (DASM) [9], rational asymmetry (RASM) [9], diagonal non-zero GC matrix (DGC) [43], differential entropy (DE) [30], power spectral density (PSD) [30], and a suite of statistical features (including mean, maximum, minimum, linear regression slope, and variance) [29]. To ensure fair comparison, all involved features were uniformly extracted on our self-built dataset ENTER and fed into the same SVM classifier for emotion recognition. A comparison of the results with those for existing methods is listed in Table 3.

From Table 3, we notice that the proposed SG_SC exhibits a marked advantage over the methods reported in the existing literature, thereby highlighting its outstanding discriminative power for emotion recognition. Its superiority can be attributed to: (1) the utilization of EEG source signals, which effectively alleviate the influence of the volume conduction effect; (2) the development of a novel EEG–fNIRS coupled brain network, which leverages the complementarity and coupling relationship of EEG and fNIRS signals.

## 5. Conclusions

In this paper, we proposed an innovative hybrid brain network in the source space as method of emotion recognition based on concurrent EEG–fNIRS signals. We initially imposed source localization on the EEG to obtain the EEG source signals, and then causal brain networks, constructed by Granger causality analysis, and coupled brain networks, constructed by coupling strengths, are integrated to constitute hybrid brain networks in the source space for emotion recognition. Through the benefits of the utilization of EEG source signals and the complementary fusion of the EEG–FNIRS dual-mode signals, the proposed method achieves recognition accuracies of 96.6% for SVM and 91.7% for KNN in the classification task for four emotions. Extensive qualitative and quantitative results reveal the superiority of the proposed method. Moreover, our method provides a novel perspective on EEG–fNIRS fusion, taking advantage of their complementarity and intrinsic relationship. However, in this paper, the source localization is relatively time-consuming, and only the neurovascular coupling relationship between EEG and fNIRS is investigated. Future work will focus on optimizing the source localization process and exploring the causality between EEG and fNIRS, which may contribute to better efficiency and performance for emotion recognition.

## Figures and Tables

**Table 1:** Emotion recognition accuracies (%) based on different brain network features.

Method	Feature	Classifier	Calm	Fear	Happiness	Sadness	Accuracy
Causal brain network	EG	SVM	86.9	84.6	85.1	87.1	86.0 ± 6.54
		KNN	75.9	71.4	73.1	67.2	71.9 ± 8.78
	SG	SVM	94.8	94.1	91.6	95.8	94.1 ± 3.32
		KNN	84.2	79.8	77.0	88.7	82.5 ± 6.30
Coupled brain network	EC	SVM	73.1	74.3	76.4	77.8	75.4 ± 7.04
		KNN	70.5	74.0	74.1	75.5	73.5 ± 8.51
	SC	SVM	81.0	79.7	78.4	81.6	80.2 ± 5.12
		KNN	82.8	83.6	82.9	86.2	83.9 ± 4.73
Hybrid brain network	EG_EC	SVM	91.5	90.1	90.8	92.6	91.3 ± 4.79
		KNN	81.1	80.5	82.0	81.4	81.3 ± 7.10
	SG_SC (ours)	SVM	97.1	96.6	95.4	97.5	96.6 ± 2.08
		KNN	91.7	90.6	89.3	95.0	91.7 ± 3.02

**Table 2:** The detailed information for the three datasets.

Dataset	Signal Type	No. Subjects	No. Trails	No. Channels	Emotion Type
ENTER	EEG/fNIRS	50	60	62/18	happiness, sadness, fear, calm
SEED-IV	EEG	15	24	62	happiness, sadness, fear, neutral
DEAP	EEG	32	40	32	HAHV, HALV, LAHV, LALV

**Table 3:** Comparison of the results with those for existing methods.

Features	Accuracy (%)
**EEG:** DASM, RASM [9]	90.7
**EEG:** DGC [43]	88.5
**EEG:** DE; **fNIRS:** statistical features [29]	90.4
**EEG:** DE, PSD; **fNIRS:** DE, PSD, mean [30]	91.8
**EEG–fNIRS**: SG_SC	96.6

## Data Availability

The data presented in this study are available on request from the corresponding author due to privacy and ethical reasons. If you are interested in the dataset and want to use it, please download and fill out the license agreement (https://gitee.com/tycgj/enter) and send it to chenguijun@tyut.edu.cn. We will send you a download link through email after a review of your application.
